# Optimal Energy Resources Allocation Method of Wireless Sensor Networks for Intelligent Railway Systems

**DOI:** 10.3390/s20020482

**Published:** 2020-01-15

**Authors:** Sheng Bin, Gengxin Sun

**Affiliations:** School of Data Science and Software Engineering, Qingdao University, Qingdao 266071, China; sungengxin@qdu.edu.cn

**Keywords:** wireless sensor network, energy resources allocation, intelligent railway system, clustering optimization, partial coverage

## Abstract

The rapid increase of train speed has brought greater challenges to the safety and reliability of railway systems. Therefore, it is necessary to monitor the operation status of trains, infrastructure, and their operating environment in real time. Because the operation environment of railway systems is complex, the construction cost of wired monitoring systems is high, and it is difficult to achieve full coverage in the operation area of harsh environments, so wireless sensor networks are suitable for the status monitoring of railway systems. Energy resources of nodes are the basis of ensuring the lifecycle of wireless sensor networks, but severely restrict the sustainability of wireless sensor networks. A construction method of special wireless sensor networks for railway status monitoring, and an optimal energy resources allocation method of wireless sensor networks for intelligent railway systems are proposed in this paper. Through cluster head selection and rotating probability model, clustering generation and optimization model, and partial coverage model, the energy consumption of nodes can be minimized and balanced. The result of simulation experiment proved that the lifetime of wireless sensor networks can be maximized by the optimal energy resources allocation method based on clustering optimization and partial coverage model, based on polynomial time algorithm.

## 1. Introduction

Railway systems have become one of the main modes of transportation in the world. Therefore, the stability, reliability, and safety of railway systems are particularly important. At present, the service state monitoring of railway systems mainly relies on the combination of manual inspection, train inspection, and on-board inspection equipment. Due to the need for offline processing and analysis of monitoring data, there is a serious delay in the monitoring of sudden faults. In order to improve the real-time and reliability of railway system service status monitoring, a cable-based status monitoring and transmission system can be constructed in critical monitoring areas. Video, fiber Bragg grating, and stress-strain monitoring and transmission technologies can be used to realize on-line monitoring of railway system infrastructure. However, due to some drawbacks of cable-based communication itself, it is difficult for on-line monitoring systems based on wired communication to achieve full coverage of railway infrastructure.

Wireless sensor network (WSN) [1] has the ability to accurately perceive monitoring objects and transmit information steadily. WSN has the characteristics of easy maintenance, easy expansion, and high reliability. It can make up for the defects of railway status monitoring systems based on wired communication, and improve the efficiency of monitoring. However, the transmission bandwidth of WSN is small, so it is not suitable for application in monitoring areas with large transmission volume [2,3,4,5]. 

The main factors restricting the large-scale application of WSN in railway system status monitoring are as follows: Firstly, the energy storage capacity of wireless sensor node is low, and it is not easy to replace power supply. Its energy utilization efficiency will determine the life cycle of nodes and even the whole network, and then it will affect the sustainability and stability of railway infrastructure and its status monitoring. Secondly, the mutual relay and forwarding of data between wireless sensor nodes will increase the transmission delay of monitoring data and affect real-time monitoring. Thirdly, WSN has low communication bandwidth, and there are many kinds and large amounts of data for the objects in railway monitoring systems. The efficiency of bandwidth resources will affect the efficiency and reliability of monitoring data transmission.

In recent years, railway status monitoring systems based on wireless sensor networks have been widely used, including intelligent monitoring system for high speed railway [6], wireless automatic monitoring and early warning system for subgrade settlement of high speed railway [7], bearing temperature monitoring system for railway freight cars based on ZigBee and GPRS (General Packet Radio Service) [8]. However, in these railway systems, the location of monitoring objects is usually fixed, and the location of sensor nodes is relatively fixed; they have not fundamentally solved the application of wireless sensor network in the railway service status monitoring system. It is necessary to optimize the allocation of limited energy and bandwidth resources in the sensor network from the routing protocol layer and data processing layer, so as to enhance the efficiency and capability of the wireless sensor network of the railway status monitoring system.

Because the battery capacity of sensor nodes is very limited, a large number of nodes will be distributed in the monitoring area to rotate for prolonging the network life cycle. Therefore, how to select the optimal nodes and effectively cover the monitoring area with the least working nodes under the condition of ensuring a certain coverage rate has become an important research field in wireless sensor network of the railway status monitoring system. Thorvaldsen et al. [9] proposed the optimal full coverage algorithm under the condition that the locations of sensors are known—it is used to realize the optimal configuration of network nodes under full coverage. However, full coverage is sometimes not necessary. For some application scenarios, only a part of the nodes to achieve partial coverage can better meet the needs. Li et al. [10] considered the problem of partial coverage and emphasized that proper partial coverage saves more energy than full coverage. Mostafaei et al. [11] focused on partial coverage, and present an efficient algorithm based on learning automata that aims at minimizing the number of sensors to activate. Habib et al. [12] proposed a greedy heuristic algorithm to deal with the coverage problem of heterogeneous WSNs in the case when full coverage of the network is not needed.

In this paper, construction method of special wireless sensor network for railway status monitoring is proposed. Based on the deployment characteristics of railway monitoring and wireless communication system, a double-layer communication network based on wireless sensor network is constructed; it is composed of monitoring sub-network layer and transmission backbone network layer. Then an optimal energy resources allocation method of monitoring sub-network for intelligent railway systems is proposed. From routing optimization and data fusion rate optimization, the method is studied to maximize the lifetime of the monitoring sub-network and enhance the ability to perceive and transmit the service status information of railway systems. Because of the status monitoring of railway systems, only partial coverage can meet the demand; at the same time, it can save energy and prolong network life. Therefore, a new partial coverage model of wireless sensor network is proposed.

The rest of the paper is organized as follows: Section 2 presents the methodology. In Section 3 we give experiments and results analysis. The last section is a summary with conclusions.

## 2. Methodology

### 2.1. Construction Method of Special Wireless Sensor Network for Railway Status Monitoring

The existing railway status monitoring system is mainly based on air and space integration technology. It combines the traditional ground monitoring system, on-board monitoring system, and vehicle-ground communication system, and joins the space monitoring and transmission system to realize the real-time wide-area monitoring of railway system. Although the railway status monitoring system based on air and space integration technology provides the richest and most comprehensive field data, as it is a complete closed system, the failure of any link may lead to the deterioration of the overall operation state of the system, and even lead to traffic safety accidents. However, wireless sensor network technology has the characteristics of easy installation, maintenance, expansion, and networking. It is more suitable for the construction of special wireless sensor network for railway status monitoring and real-time monitoring of the operation status of the system.

The monitoring objects of the special wireless sensor network for railway status monitoring are mainly railway infrastructure and its operating environment, which are all deployed along railway tracks, so they are approximately linear or banded.

**Figure 1:**
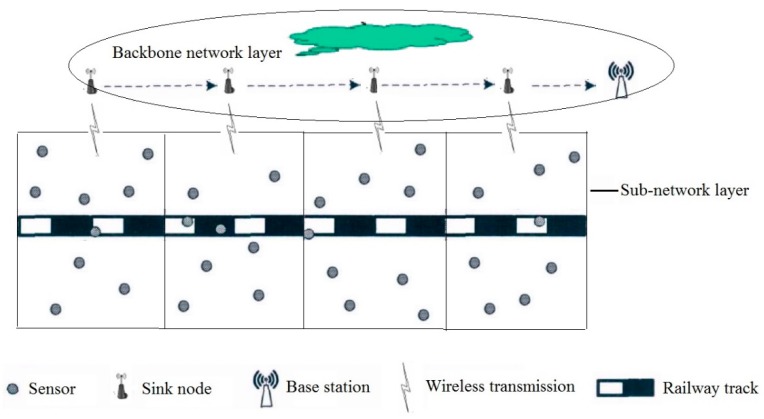
Architecture of double-layer wireless sensor network for railway status monitoring system.

The main function of special wireless sensor network is to sense the service status of railway infrastructure and its operating environment and send monitoring data to the base station near the monitoring area. Therefore, the deployment location of wireless sensor nodes and infrastructure correspond to each other. Wireless sensor nodes have limited energy storage, and their communication energy consumption is mainly determined by the amount of monitoring data and its transmission distance. At the same time, the communication distance of wireless sensor nodes is limited, so it is not feasible for the sensor nodes in the monitoring area to communicate directly with the base station. Therefore, the method divides the whole monitoring area into several sub-monitoring areas, and deploys a sink node at the edge of each sub-monitoring area to receive the monitoring data of sub-monitoring area, while the data in all sub-monitoring areas is forwarded from sink node to base station. On the one hand, the method can ensure that the data perceived by each sensor node can be sent to the base station smoothly; on the other hand, it also greatly reduces the energy consumption of sensor nodes and improves the life cycle of special wireless sensor networks. The architecture of double-layer wireless sensor network for railway status monitoring system is shown in Figure 1.

As shown in Figure 1, the special wireless sensor network for railway status monitoring is a double-layer structure, which consists of sub-network layer and transmission backbone network layer. The sub-network layer consists of several sub-monitoring areas. The wireless transmission network in each sub-monitoring area consists of several wireless sensor nodes and one wireless sink node. The sink node is responsible for collecting the sensing data of all sensor nodes in the sub-areas. The backbone network layer consists of several wireless sink nodes and a base station. The sink node is responsible for forwarding the data collected from the subnet layer to the base station. The service status data of railway infrastructure and its operating environment is first perceived by wireless sensor nodes in the sub-network, then the sensor nodes in each sub-monitoring area send the sensing data to the corresponding sink node. Finally, the sink node sends all the sensing data to the base station, and then the base station sends it to the remote server for the diagnosis and prediction of the status of railway system.

**Figure 2:**
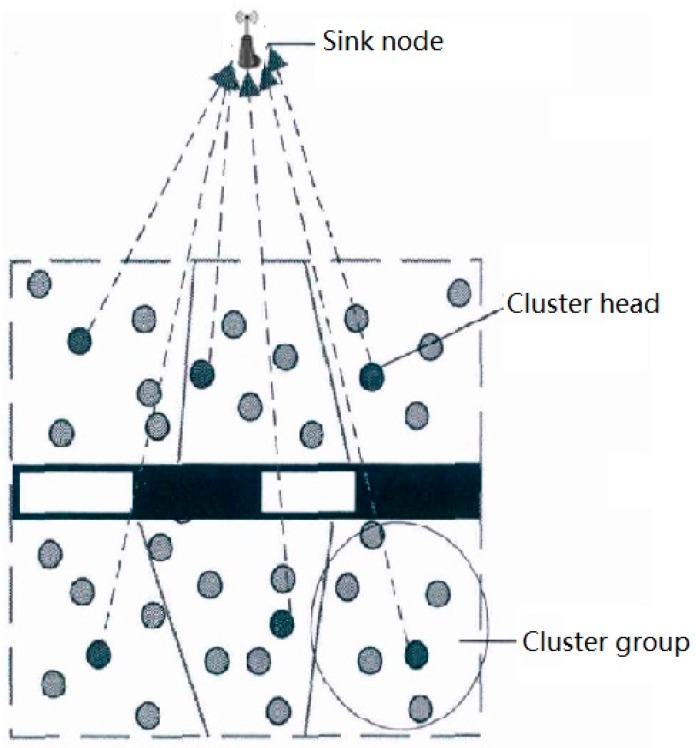
Structure of sub-monitoring area.

As shown in Figure 2, in sub-monitoring area, sensor nodes are responsible for sensing service status data of infrastructure and its operating environment on the one hand, and sending sensing data to sink nodes on the other hand. Because the energy storage of sensor nodes is limited, the energy efficiency of sensor nodes is mainly considered in routing protocol selection and optimization.

The communication energy consumption of wireless sensor nodes is mainly determined by the amount of data transmitted and the transmission distance. As can be seen from Figure 2, the distances between sensor nodes and sink nodes are different. When each sensor node transmits the same amount of data to sink nodes, the energy consumption of each sensor node will be unbalanced, and the nodes far away will fail prematurely due to energy exhaustion.

In order to improve the energy efficiency of sensor nodes in the sub-network, cluster communication routing protocol [13] is adopted in this system. In a sub-network, one node is selected as the cluster head according to the needs in the cluster, and the other nodes are the members of the cluster. Cluster members are responsible for the data perception of key components of the railway system and send the perception data to cluster head nodes. After receiving the data, the cluster head node carries out simple processing and analysis, and forwards the data to the sink node. By using clustering communication routing protocol, the choice of cluster head and the result of clustering have a great influence on the total energy consumption of all nodes and the balance of energy consumption among nodes. In addition, the communication energy consumption of wireless sensor nodes is also related to the amount of data transmitted. Cluster heads collect data from cluster members and then process data before sending. This can reduce the amount of data transmitted and corresponding communication energy consumption, but at the same time it will increase the corresponding data processing energy consumption. The adjustment and balance between them can effectively reduce the total energy consumption of cluster head nodes and improve the overall energy utilization efficiency of the sub-network. In order to improve the energy efficiency of nodes in the sub-network and the lifecycle of the sub-network, the optimization methods of cluster communication routing protocol and data fusion efficiency of cluster head nodes will be studied.

### 2.2. An Optimal Energy Resources Allocation Method Based on Clustering Optimization

The sub-network layer of railway wireless monitoring network is mainly composed of several sensor nodes and a sink node. Sensor nodes are responsible for sensing, processing, and short-distance transmission of data, while sink nodes are responsible for collecting data perceived by all sensor nodes in the monitoring sub-network. Sensor nodes are usually composed of four parts: Data sensing unit, data processing unit, data communication unit, and energy supply unit. Data sensing unit is responsible for perceiving real-time service status data of monitoring objects. Data processing unit is responsible for simple format transformation of perceptual data and other processing work. The data communication unit is responsible for data communication with other sensors or sink nodes, including the process of receiving and transmitting data. The energy supply unit is responsible for energy supply for the remaining three units to ensure the stability of each unit.

The energy consumption of wireless sensor nodes is mainly in three stages: Data sensing, data processing, and data transmission. According to the literature [14], the energy consumed by sensors in the process of data sensing is negligible compared with the other two stages. Therefore, the energy consumption of sensor nodes in the process of data processing and communication when establishing the energy consumption model of railway wireless monitoring sub-network.

**Figure 3:**
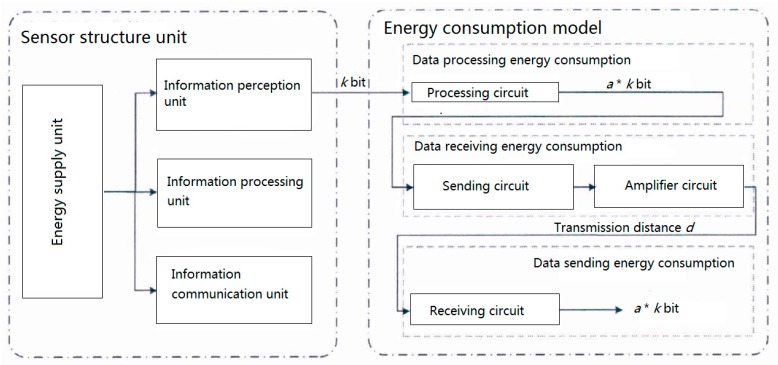
Composition structure and energy consumption of wireless sensor nodes.

As shown as Figure 3, the energy consumption of sensor nodes in data communication process consists of three parts: Data processing energy consumption, data receiving energy consumption, and data sending energy consumption [15,16].

The energy consumption of data processing is mainly determined by the energy consumption rate of data processing and the amount of data processed, as shown in Equation (1):

(1) $$E_{X}\left(l\right)=l\times E_{A}$$

where l represents the amount of data processed, $E_{A}$ represents energy consumption of data processing circuits for processing unit data.

The energy consumption of data reception is mainly determined by the amount of data received, as shown in Equation (2):

(2) $$E_{R}\left(l\right)=l\times E_{C}$$

where l also represents the amount of data processed, $E_{C}$ represents energy consumption of transmitting circuit.

The energy consumption of data transmission is mainly related to the amount of data sent and the distance sent, as shown in Equation (3):

(3) $$E_{T}\left(l,d\right)=\{\begin{array}{c} E_{R}\left(l\right)+l\times E_{f}\times d^{2},\;(d<d_{0})\\ E_{R}\left(l\right)+l\times E_{m}\times d^{4},\;\left(d\geq d_{0}\right) \end{array}$$

where d represents the distance between two nodes, $d_{0}$ is a constant, the selection of its value is related to network environment. When the distance between sending node and receiving node is less than $d_{0}$ in the network, energy dissipation model in free space [17] is adopted, the energy consumption of sending data is proportional to the square of distance. $E_{f}$ represents the energy consumption coefficient of free space power amplifier. When the distance between sending node and receiving node is greater than or equal to $d_{0}$ in the network, multi-channel attenuated energy dissipation model [18] is adopted, the energy consumption of sending data is proportional to the fourth power of distance. $E_{m}$ represents the energy consumption coefficient of multichannel attenuated power amplifier.

The sink node in the subnet is mainly responsible for receiving information, and the energy storage of the sink node is much larger than that of the other sensor nodes. Therefore, in this chapter, it is assumed that the energy of the sink node is infinite.

In order to reduce the energy consumption of sensor nodes, a routing protocol based on clustering communication is used to transmit data in the sub-network. Firstly, all sensors in the sub-network are divided into several clusters, and then a node in each cluster is selected as the cluster head, which is responsible for collecting the data of the remaining sensor nodes in the cluster and forwarding the data to the corresponding sink node. Therefore, the communication load and corresponding energy consumption of cluster head nodes are much higher than those of other non-cluster head nodes. In order to ensure the balance of energy consumption of each node and avoid the failure of cluster head nodes due to excessive energy consumption, it is necessary to re-select and rotate the cluster head before each round of communication. The data between cluster head and cluster members is transmitted by single-hop forwarding, and the data transmission between cluster head and sink node is also carried out by single-hop forwarding.

**Figure 4:**
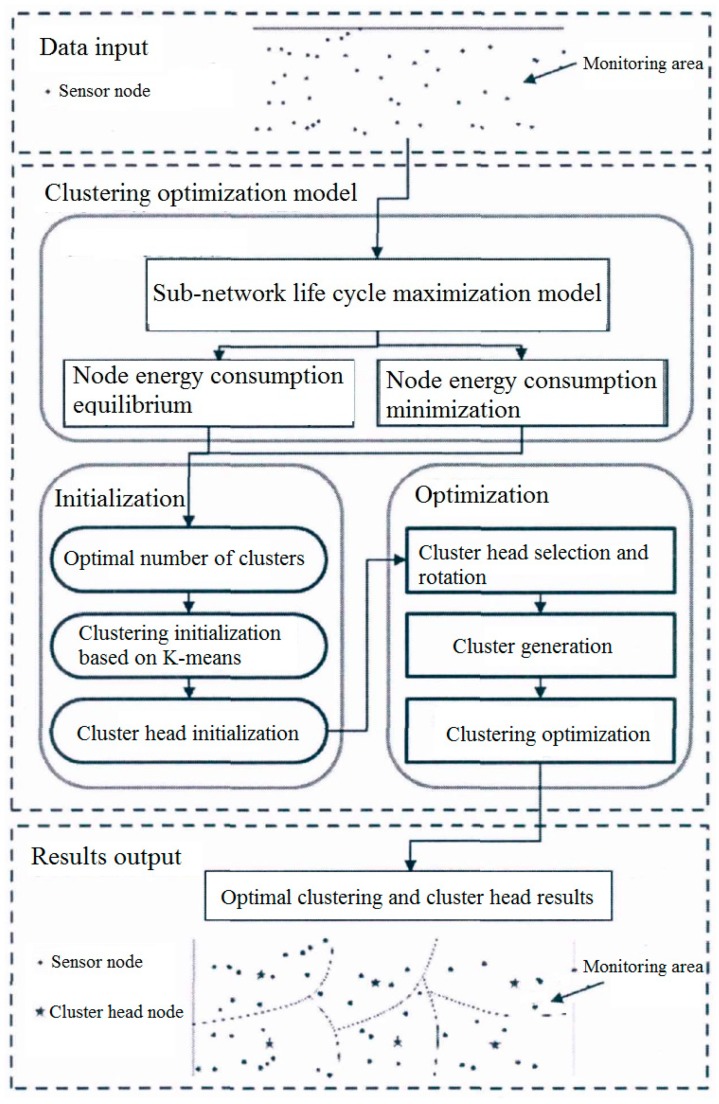
Structure of cluster-based energy optimization routing protocol for sub-network.

The structure of cluster-based energy optimization routing protocol for sub-network is shown in Figure 4.

The structure of cluster-based energy optimization routing protocol consists of three layers: Data input layer, clustering optimization strategy layer, and data output layer. 

The data input layer is mainly responsible for inputting the initial data of each sensor node in the monitoring sub-network into the target model for optimizing the processing. The input data mainly includes the number of sensors located around the track, energy storage, and location information.

In clustering optimization strategy layer, sensor networks and their nodes aim at minimizing and balancing overall energy consumption, constantly updating the selection of all clusters and their cluster heads, so as to enhance the life cycle of railway status monitoring systems. In this paper, an efficient optimization model to optimize the cluster generation and cluster head selection is proposed; it can ensure that the sub-network always works in the mode of the highest energy efficiency. In the process of cluster head selection and rotation, a comprehensive index considering the candidate probability, residual energy, and predicted energy consumption of candidate cluster head nodes is proposed. Then, a multi-objective optimization model is constructed, and genetic algorithm is used to optimize and update the formation of clusters.

In the data output layer, all sensor nodes in the sub-network are allocated to different clusters for data transmission in an energy-efficient optimization way, which would improve the life cycle of the monitoring sub-network.

#### 2.2.1. Clustering and Cluster Head Initialization Based on Improved K-Medoids

K-medoids algorithm can cluster all nodes and distribute the nodes with similar distances into a unified cluster. However, the initial cluster centers of K-medoids algorithm are randomly selected. The clustering effect depends on the selection of the initial cluster centers. Unbalanced distribution of cluster centers will lead to local optimum clustering results. Therefore, the improved K-medoids algorithm is proposed to cluster in this paper. It ensures that the initial cluster centers are distributed as evenly as possible in the whole monitoring area, and improves the global optimal performance of the clustering results. In addition, when the improved K-medoids algorithm is used to optimize clustering based on genetic algorithm, its optimization speed and effect are improved significantly.

When improved K-medoids algorithm is used to initialize clusters, the optimal number of clusters must be determined firstly. When the number of clusters is too small, the number of cluster members increases correspondingly, which will greatly increase the communication burden and energy consumption of cluster head nodes. When the number of clusters is too large, most sensor nodes and sink nodes transmit data directly, and the clustering optimization algorithm will become meaningless. So, it is necessary to optimize the number of clusters with the objective of minimizing the total energy consumption of all nodes in the sub-network.

Suppose that *T* sensor nodes are evenly located in the monitoring sub-network area of N×N, where x∈[−N/2,N/2], y∈[−(1+ε)N,−εN], ε is a distance adjustment parameter, it is used to adjust the vertical distance between monitoring sub-network area and sink node. The probability density of distribution is $S\left(x,y\right)=1/M^{2}$. The sink node is located at the origin of coordinates.

All sensors distributed in the sub-network are divided into cluster head nodes and cluster member nodes, so the total energy consumption of sub-network nodes is also composed of two parts as follows:

(4) $$E_{total}=\overset{k}{\underset{i=1}{\sum }}\left(E_{C}^{i}+\overset{n-1}{\underset{j=1}{\sum }}E_{Non-C}^{ji}\right)$$

where $E_{C}^{i}$ represents energy consumption of the *i*-th cluster head node, $E_{Non-C}^{ji}$ represents energy consumption of the *j*-th non-cluster head node in the *i*-th group, n represents the number of sensor nodes within each cluster, k represents the number of clusters. Assuming that *T* nodes within the sub-network are evenly allocated to each cluster, there are n=T/k nodes in each cluster, including one cluster head and *T* − 1 non-cluster head nodes.

When all nodes have enough energy to receive, process, and send all data in the cluster with the role of cluster head. However, the residual energy of sensor nodes decreases with the increase of communication rounds. When the energy of all nodes in a cluster is below a certain threshold, it is not enough to send all data in the cluster to the sink node as the cluster head. In this paper, the number of sensor nodes in each cluster and the amount of data transmitted by cluster head will be reduced by increasing the number of clusters, effectively reducing the energy consumption of cluster head so that the communication of the system can be restored and continued.

In this paper, based on the optimal cluster groups, an improved K-medoids algorithm is used to initialize the clusters. According to the distance information of all sensor nodes in the sub-network, it is allocated to *K* clusters. The specific steps of cluster initialization based on improved K-medoids algorithm (Algorithm 1) are defined as follows:
**Algorithm 1 Cluster initialization based on improved K-medoids algorithm**Input: Location and energy information of *T* sensor node in in the monitoring sub-network area of N×N
Output: *T* sensor nodes are allocated to *K* clusters in the mode of optimal energy consumptionStep 1: Determining the optimal clustering group number $k_{opt}$;Step 2: Selecting a sensor node as the first cluster center randomly;Step 3: Calculating the distance from each node to the selected cluster center;    **repeat**      **for**
*i* = 1, 2, …, *k*        **for**
*j* = 1, 2, …, *T* − *k*          $d_{CtoC_{ji}}=\sqrt{{\left(x\left(j\right)-x\left(i\right)\right)}^{2}+{\left(y\left(j\right)-y\left(i\right)\right)}^{2}}$
    **until** the distances between all nodes are calculatedStep 4: Selecting the *k*-th ($1<k<k_{opt}$) clustering center    **repeat**      **for**
*j* = 1, 2, …, *T* − *k*        **for**
*i* = 1, 2, …, *k*          $d_{CtoC_{j}}=\min\left(d_{CtoC_{ji}}\right)$;                $\mathrm{sum}\left(d\left(x_{CtoH_{j}}\right)\right)=\overset{T-k}{\underset{j=1}{\sum }}d_{CtoC_{j}}$
                  $p_{j}=\frac{d\left(x_{CtoH_{j}}\right)}{\mathrm{sum}\left(d\left(x_{CtoH_{j}}\right)\right)}$
      **until** all $k_{opt}$ cluster centers had been selectedStep 5: Initialization of all clusters based on selected $k_{opt}$ cluster centers    **repeat**        **for**
*i* = 1, 2, …,$k{\;}_{opt}$          **for**
*j* = 1, 2, …, *T* − $k_{opt}$
              $d_{CtoC_{ji}}=\sqrt{{\left(x\left(j\right)-x\left(i\right)\right)}^{2}+{\left(y\left(j\right)-y\left(i\right)\right)}^{2}}$
            **for**
*j* = 1,2,…,*T*-$k_{opt}$             **for**
*i* = 1, 2, …, $k_{opt}$              $d_{CtoC_{j}}=\min\left(d_{CtoC_{ji}}\right)$;    **until** all sensor nodes are allocated to the cluster with the lowest energy consumption.

In Step 4, the node far from the selected cluster center is more likely to be selected as the new cluster center, which can ensure the decentralization of the cluster center in the whole monitoring area. In Step 5, on the basis of determining the cluster center, each sensor chooses to join the cluster corresponding to the nearest cluster center, thus the initialization of the cluster would be completed.

In the process of assigning all sensor nodes in the sub-network to *K* clusters by using the improved K-medoids algorithm, the cluster center is not the final cluster head. Therefore, after initialization of cluster, in order to minimize the total energy consumption of cluster members and cluster heads, the node with the smallest sum of distances from all nodes in the cluster is chosen as the cluster head. According to Equation (5), the position of virtual cluster head (the center of all nodes) is calculated, and then the nearest node to the virtual cluster head is selected as the initial cluster head node.

(5) $$Virtual_{H\left(x\left(k\right),y\left(k\right)\right)}=\left(\frac{\sum_{i=1}^{n_{k}}x\left(i\right)}{n_{k}},\frac{\sum_{i=1}^{n_{k}}y\left(i\right)}{n_{k}}\right)$$

Compared with other existing methods, cluster initialization based on improved K-medoids algorithm can divide the nodes with similar distance into the same cluster, which can reduce the energy consumption of information transmission between cluster members and cluster heads, and greatly improve the life cycle of the network.

#### 2.2.2. Cluster Head Selection and Rotating Probability Model

In the routing protocol of cluster communication, the energy consumption of cluster head node is much larger than that of non-cluster head node. Therefore, the cluster head should be re-selected before each round of communication. It could effectively guarantee the energy balance among sensor nodes and avoid the failure of cluster head node due to excessive energy consumption. In the cluster head selection and rotating probability model, the candidate probability of cluster head, the residual energy rate of nodes, and the energy consumption rate of cluster head prediction are considered.

In order to maintain the balance of energy consumption among nodes, each node in the cluster should have a relatively equal opportunity to serve as cluster head. That is to say, the *T*/*k* cluster members in the cluster have a chance to act as cluster head in the next *T*/*k* round communication process [19,20].

If the sensor node in the subnet has served as cluster head in the previous *r*-1 round of communication, then the node will not participate in the cluster head election in the *r*-th round, so the candidate probability model of cluster head is defined as:

(6) $$p_{1i}\left(r\right)=\{\begin{array}{c} k/T-k\times r,\;C_{i}\left(r\right)=1\\ 0,\;C_{i}\left(r\right)=0 \end{array}$$

where r∈[1,T/k] represents that when all the nodes in the cluster have served as the cluster head once in the previous communication process, r will be reset to 1, then it gradually grows to T/k. $C_{i}\left(r\right)$ represents role state of node *i* in the past *r* round communication. If it had served as a cluster head, then $C_{i}\left(r\right)=0$, otherwise, $C_{i}\left(r\right)=1$.

After multiple rounds of communication, the residual energy of each sensor node in the sub-network will be different from each other. In the process of cluster head selection and rotation, the probability that nodes with more residual energy are selected as cluster heads is higher. The residual energy probability model of each node is defined as follows.

(7) $$p_{2i}\left(r\right)=\frac{\left|E_{R}^{i}\left(r\right)-E_{R}^{min}\left(r\right)\right|}{\sum_{i=1}^{n}\left|E_{R}^{i}\left(r\right)-E_{R}^{min}\left(r\right)\right|}$$

where $E_{R}^{i}\left(r\right)$ represents residual energy of the *i*-th node in the *r*-th round, $E_{R}^{min}\left(r\right)$ represents minimum residual energy of intra-cluster nodes.

The residual energy of the *i*-th node in the *r*-th round is calculated as follows.

(8) $$E_{R}^{i}\left(r\right)=E_{R}^{i}\left(r-1\right)-E_{Co}^{i}\left(r-1\right)$$

where $E_{Co}^{i}\left(r-1\right)$ represents energy consumption of nodes in (*r* − 1)-th round. 

If the node acts as cluster head in the (*r* − 1)-th round, its energy consumption can be calculated by Equations (9)–(12).

(9) $$E_{Co-CH}\left(r-1\right)=E_{R}\left(r-1\right)+E_{D}\left(r-1\right)+E_{T}\left(r-1\right)$$

(10) $$E_{R}\left(r-1\right)=\left(n_{k}-1\right)\times l\times E_{C}$$

(11) $$E_{D}\left(r-1\right)=n_{k}\times l\times E_{A}$$

(12) $$E_{T}\left(r-1\right)=l\times E_{C}+l\times \vartheta_{m}\times d_{t}^{4}\left(r-1\right)$$

where $\vartheta_{m}$ represents energy consumption coefficient of power amplifier based on multipath attenuation model, $d_{t}$ represents distance from cluster head node to sink node.

If a node acts as a non-cluster head node in the (*r* − 1)-th round, its energy consumption can be calculated as follows.

(13) $$E_{Co-NCH}\left(r-1\right)=l\times E_{C}+l\times \vartheta_{f}\times d_{t}^{2}\left(r-1\right)$$

where $\vartheta_{f}$ represents energy consumption coefficient of power amplifier based on free space model.

If a node has a lot of residual energy, but it consumes a lot of energy when it is chosen as cluster head node, and even fails because of the exhaustion of energy after it is chosen as cluster head, such a node is not suitable for serving as cluster head. Therefore, in the process of cluster head selection and rotation, the energy consumption of nodes as cluster heads is predicted and compared, and higher priority probability is given to nodes with less energy consumption. The predicting energy consumption rate model of cluster head nodes in (*r* + 1)-th round is defined as follows:

(14) $$p_{3i}\left(r\right)=\frac{\left|E_{Co}^{i}\left(r+1\right)-E_{Co}^{max}\left(r+1\right)\right|}{\sum_{i=1}^{n_{k}}\left|E_{Co}^{i}\left(r+1\right)-E_{Co}^{max}\left(r+1\right)\right|}$$

where $E_{Co}^{i}\left(r+1\right)$ represents the predicting energy consumption of node in *r* + 1 round after serving as cluster head, $E_{Co}^{max}\left(r+1\right)$ represents the predicting maximum energy consumption of all nodes as cluster heads.

Considering the above three factors, the comprehensive probability model is defined as:

(15) $$p_{i}\left(r\right)=\mu_{1}\times p_{1i}\left(r\right)+\mu_{2}\times p_{2i}\left(r\right)+\mu_{3}\times p_{3i}\left(r\right)$$

where $\mu_{1}$, $\mu_{2}$ and $\mu_{3}$ are weight coefficients, which are used to adjust the influence of various factors on the comprehensive model.

#### 2.2.3. Clustering Generation and Optimization Model

After the cluster head is determined, the system will optimize the cluster with the goal of minimizing the total energy consumption and balancing the energy consumption among nodes, so as to maximize the lifetime of the special wireless sensor network sub-network. By optimizing the number of cluster members and the corresponding relationship between cluster head and cluster members, the minimization of energy consumption is taken into account under the premise of ensuring the balance of energy consumption, effectively guaranteeing the maximization of sub-network life cycle.

The energy consumption of cluster head node is determined together by the distance between cluster head and sink node and the number of cluster members. However, the distance between cluster head and sink node varies with the choice of cluster head. Therefore, the number of cluster members should also change to ensure the energy balance between cluster head nodes. The cluster size optimization model is defined as follows:

(16) $$f_{1}\left(E_{Ci}\left(n_{i}\right)\right)=\min\frac{\sum_{i=1}^{k}{\left(E_{Ci}\left(n_{i}\right)-\bar{E_{C}}\right)}^{2}}{k}$$

where the objective of $f_{1}\left(E_{Ci}\left(n_{i}\right)\right)$ is to minimize the variance of energy consumption among all cluster heads, it represents the equilibrium of energy consumption of each cluster head. $E_{Ci}$ represents cluster head energy consumption of *i*-th cluster, $\bar{E_{C}}$ represents mean energy consumption of cluster heads for all clusters, k represents the number of clusters and cluster heads.

After cluster size optimization, the energy consumption among cluster heads is basically balanced, while the cluster size optimization model only determines the number of members in each cluster, and it does not determine the corresponding relationship between non-cluster head nodes and cluster heads. However, when the same cluster member node is assigned to different clusters, the communication energy consumption varies with the distance from the cluster head node. In order to minimize the communication energy consumption of non-cluster head nodes, the corresponding relationship between cluster head and cluster members needs to further optimize. The total energy consumption model for all nodes is defined as:

(17) $$f_{2}\left(E_{Cj},E_{non-Ci}\right)=\min\left(\overset{T-k}{\underset{i=1}{\sum }}E_{non-Ci}+\overset{k}{\underset{j=1}{\sum }}E_{Cj}\right)$$

where the objective of $f_{2}\left(E_{Cj},E_{non-Ci}\right)$ is to minimize the total energy consumption of all nodes, $E_{non-Ci}$ represents energy consumption of the *i*-th non-cluster head node, $E_{Cj}$ represents energy consumption of the *j*-th cluster head.

The optimization of cluster size is used to balance the energy consumption among cluster head nodes, while the optimization of the corresponding relationship between cluster head and cluster members can effectively reduce the total energy consumption of non-cluster head nodes. At the same time, the optimization of the corresponding relationship between cluster head and cluster members will affect the optimization of cluster size in turn. Therefore, the optimization of cluster size and the corresponding relationship between cluster head and cluster members is a multi-objective optimization problem, which can be summed up as a kind of problem which can make multiple objectives reach optimal simultaneously under certain constraints. For example, in order to select the appropriate cutting speed and feed ratio in mechanical processing, the objectives are put forward: (1) The lowest machining cost, (2) the highest productivity, and (3) the longest cutter life.

The comprehensive optimization model of node clustering is defined as:

(18) $$\min\;f\left(E\right)=\left(f_{1}\left(E_{Ci}\left(n_{i}\right)\right),f_{2}\left(E_{Cj},E_{non-Ci}\right)\right)$$

There are two constraints which must be satisfied for clustering multi-objective optimization model: The first constraint is that the total number of nodes in each cluster should be at least two, and the second constraint is that failed sensor nodes do not participate in the formation and optimization of a new round of node clustering. 

Clustering formation and optimization process is a non-linear multi-objective optimization problem [21]. Therefore, in the actual optimization process, heuristic optimization algorithm is often used to solve multi-objective optimization problems. In this paper, genetic algorithm [22] is used to solve the multi-objective optimization model.

The optimization algorithm based on genetic algorithm is a process of getting the optimal chromosome through chromosome selection, crossover, mutation, and other processes based on the initial population. In this paper, the population size (the number of chromosomes) is *m*, each chromosome consists of *N* genes. The position and content of each gene respectively represent the position of the sensor and the corresponding cluster number to be assigned.

**Figure 5:**
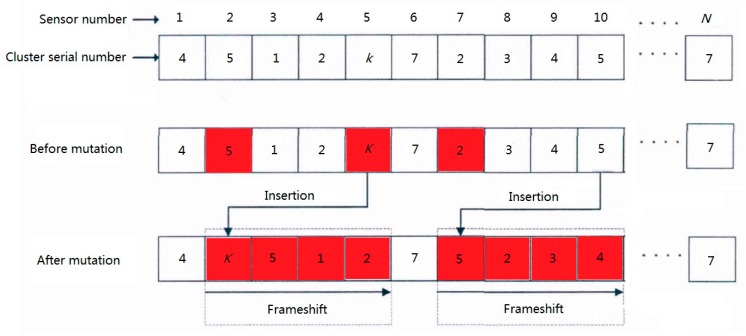
Chromosome mutation process.

As shown Figure 5, the sensors of the railway status monitoring system are all assigned with fixed numbers [1, 2, …, *N*], and the cluster numbers [1, 2, ..., *k*] indicate the corresponding cluster serial numbers of each sensor.

Genetic algorithm needs many iterations to get the optimal result. In order to improve the optimization speed of genetic algorithm, K-medoids algorithm is used to initialize clustering, and an initial chromosome close to the optimal result is generated in this paper. Then, on the basis of clustering chromosomes, the remaining *m* − 1 chromosomes are generated by the way of frameshift mutation. All *m* chromosomes together constitute the initial parent population *P*.

The specific steps for solving multi-objective optimization of clustering by genetic algorithm (Algorithm 2) are defined as follows:
**Algorithm 2 Multi-objective optimization of clustering by genetic algorithm**Input: Population size *m*, iteration times *T*Output: Optimal clustering results (the optimal chromosomes)Step 1: Generation of *m* chromosomes and formation of initial paternal population *P* based on improved K-medoids algorithm;Step 2: Generation of *m* chromosomes by two-point crossover selection to form a new offspring population *Q*;Step 3: Combining the parent population *P* with the offspring population *Q* to form a new population *A*;Step 4: Non-dominant sorting;Step 5: Crowding-distance operator computing;Step 6: Based on the non-dominant sorting value and crowding-distance information, the optimal m chromosomes were selected from the new population *A*, and the parent population *P* was updated.;Step 7: If the optimal termination condition is satisfied (the optimal result is obtained or the maximum number of iterations is reached), the optimal chromosome in the optimal population is selected, and the final result of clustering for sub-network is output, otherwise go to Step 2.

### 2.3. Partial Coverage Model of Wireless Sensor Network Based on Polynomial Time Algorithm

The basic idea of wireless sensor network coverage model is to give the number of targets *T*, use the number of limited nodes *N*, limited energy *E*, and improve the lifetime of wireless sensor network on the premise of meeting the coverage requirements.

In order to ensure that any position in the monitoring area is covered by at least one sensor node at any time, the full coverage model is often adopted. In wireless sensor network coverage model, $S=\left\{s_{1},\;s_{2},\ldots ,s_{N}\right\}$ represents sensor nodes set, $R=\left\{r_{1},\;r_{2},\ldots ,r_{T}\right\}$ represents coverage targets set. The sensing range of wireless sensor nodes $s_{k}$ is a circle with sensing radius as its radius.

**Figure 6:**
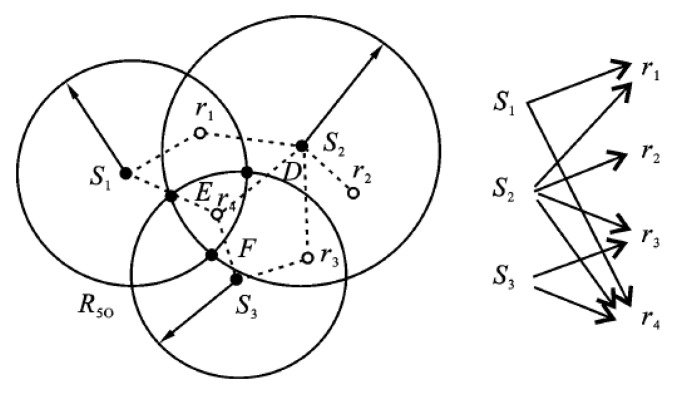
Node coverage of wireless sensor network.

An example of node coverage of wireless sensor network is shown in Figure 6.

As shown in Figure 6, target $r_{1},r_{2},r_{3},r_{4}$ receive wireless sensor node $s_{1},s_{2},s_{3}$ monitoring, respectively. The relationship between sensor node and target is not one to one; it results in the imbalance between energy consumption and residual energy of sensor nodes.

Considering the balance of energy consumption of wireless sensor network, the energy of a certain area $E_{k}$ is defined as follows:

(19) $$E_{k}=\frac{\sum_{i=1}^{N_{k}}E_{k_{i}}}{N_{k}}$$

where $E_{k_{i}}$ represents the residual energy of the *k*-th cluster, $N_{k}$ represents number of nodes in the *k*-th cluster.

However, the full coverage algorithm would produce a large number of redundant nodes and generally require nodes to have accurate location information, which generates a great challenge for constructing a low-cost and high-density wireless sensor network.

In reality, full coverage sometimes is not necessary. Partial coverage algorithm means that the deployed wireless sensor network only covers part of the monitoring area at a certain time. Under the premise of meeting the required coverage, in each round, some nodes are selected to work, and the rest nodes are in the sleep state.

In this paper, polynomial time algorithm is used to construct a new partial coverage model, which transforms the nonlinear programming problem into a linear programming problem, and the optimal solution of partial coverage can be obtained within the computable complexity.

Suppose there is a partial coverage vector $\theta =\left\{\theta_{1},\theta_{2},\ldots ,\theta_{n}\right\}$ in the whole life cycle *L*, it makes the target $r_{k}$ satisfy the mathematical relation f: There exists node $s_{k}$ which covers monitoring target $r_{k}$ at least in $L\cdot \theta_{k}$ time. If Boolean variable $y_{i,k}$ is true, it means that node $s_{k}$ meets coverage needs, and it is responsible for the monitoring of target $r_{k}$. So, the partial coverage problem can be described as follows:

(20) $$\underset{f\in F}{\sum }\underset{s_{i}\in C}{\sum }y_{i,k}\cdot t_{p}\geq L\cdot \theta_{k}$$

where the objective function $t_{p}$ is the time segment of mathematical relation f, C represents the cluster in working condition.

By optimizing Equation (5), we can summarize the analysis of partial coverage problem as follows: The objective function of partial coverage model is $T_{opt}$, the variable of the model is the total time $b_{i,k}$ of node $s_{k}$ covering target $r_{k}$. So partial coverage model can be defined as:

(21) $$\underset{}{\max}T_{opt}$$

(22) $$\underset{s_{i}\in C}{\sum }b_{i,k}\geq L\cdot \theta_{k}$$

(23) $$\underset{r_{k}\in C}{\sum }b_{i,k}\cdot e_{0}\leq E$$

(24) $$0\leq b_{i,k}\leq L,\;L\geq 0$$

For target $r_{k}$, node coverage condition is $b_{i,k}>0$. For sensor node $s_{k}$, the time length when it covers $r_{k}$ is $b_{i,k}$.

## 3. Experiments and Results

In this section, the proposed optimization model based on clustering optimization for optimal allocation of energy resources in railway wireless sensor networks is simulated and validated. By comparing with the two representative algorithms (FAF-EBRP algorithm [23] and MOFCA algorithm [24]) in terms of life cycle and the amount of data collected by sink nodes, the advantage of the proposed optimization model in energy efficiency is verified.

In the experiment, a 600-m-long track and surrounding infrastructure were selected as monitoring objects. The monitoring area can be approximated to a 600-m-long and 60-m-wide rectangular area. All sensors used for monitoring were randomly distributed in the monitoring area.

Then the monitoring area was evenly divided into 10 monitoring sub-networks, each of which was a square area of 60 m×60 m. A sink node was located in the center of each sub-network monitoring area. The sensor nodes in the sub-network transmit the monitoring data to the corresponding cluster head and sink node, and then transmit the monitoring data to the base station by the sink node. One of the sub-networks was selected as the simulation environment to verify the energy-saving effect of routing protocols. In the simulation scenario, a total of 200 sensor nodes were randomly distributed in the monitoring area by setting the track as X-axis and the endpoint far from the base station as the coordinate origin.

The simulation parameters are shown in Table 1.

**Figure 7:**
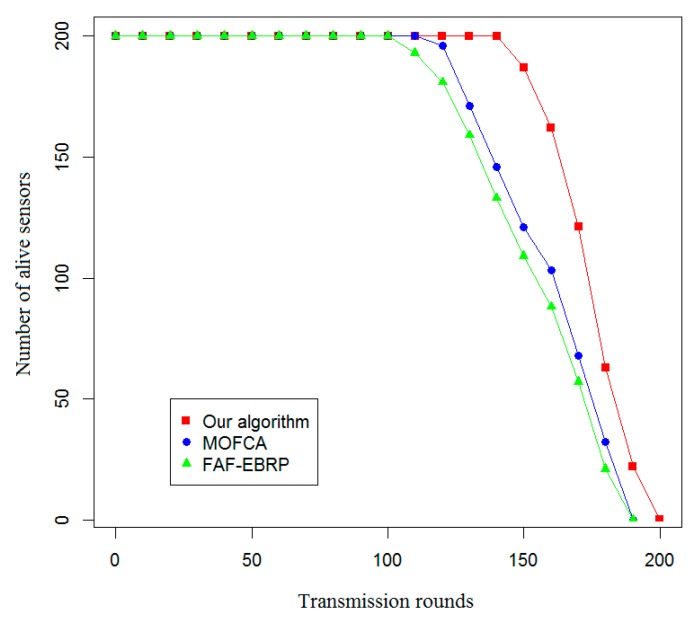
Comparison of the effective life cycle of the system.

**Figure 8:**
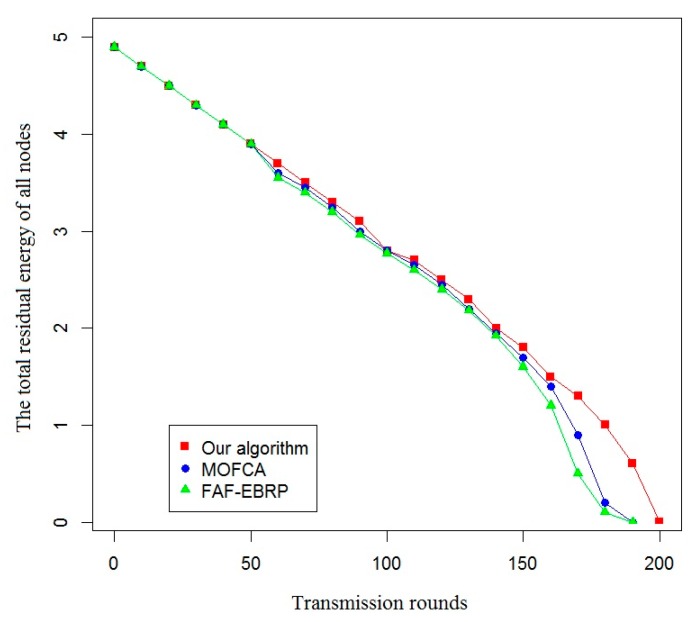
Comparison of the total residual energy of all nodes.

**Figure 9:**
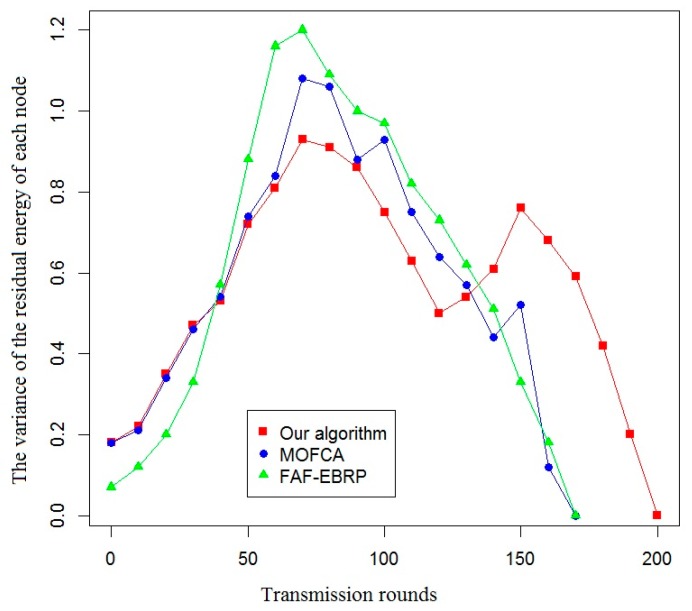
Comparison of the variance of the residual energy of each node.

**Figure 10:**
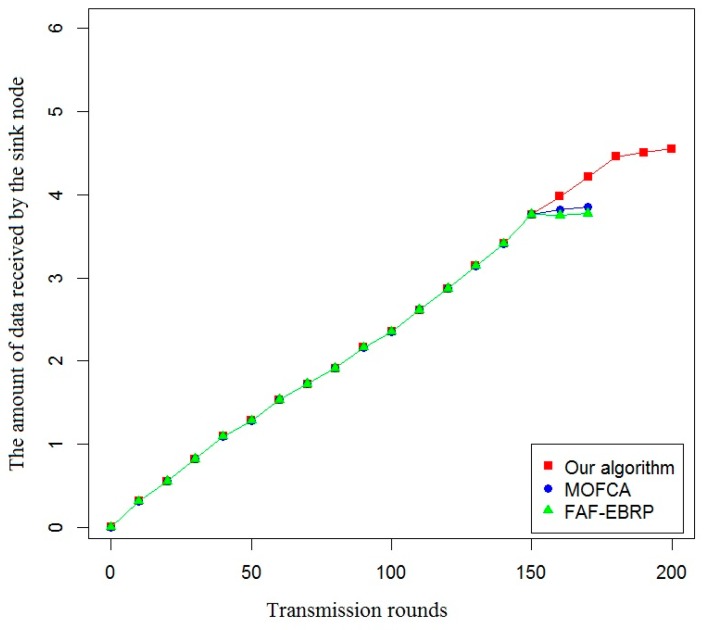
Comparison of the amount of data received by the sink node.

In this paper, the effective life cycle of the system, the total residual energy of all nodes, the variance of the residual energy of each node, and the amount of data received by the sink node were used as evaluation indexes to compare the energy efficiency of the proposed algorithm with that of the other two algorithms. The simulation results are shown in Figure 7, Figure 8, Figure 9 and Figure 10.

As shown in Figure 7, when using the proposed routing protocol to communicate, the nodes of the special wireless sensor network for railway status monitoring begin to fail due to energy exhaustion only after 200 rounds of communication. When using the other two routing protocols, the nodes of the sub-network begin to fail after 190 rounds, and the trend of node failure is similar, but the effect of MOFCA is slightly better.

In Figure 8, the total residual energy of all nodes represents the energy-saving effectiveness of different routing protocols. When using these three protocols, the curve of total residual energy changes smoothly, but based on the proposed protocol in this paper, the total residual energy of nodes is more, and the rounds of communication are more.

As shown in Figure 9, the variance of residual energy of each node is smaller than that of the other two protocols when using our routing protocol. It means that the energy consumption of each node in the sub-network is more balanced, and it is more conducive to improving the overall effective life cycle of the system.

As shown in Figure 10, the proposed routing protocol can ensure that the sink node receives more information about the service status of railway infrastructure and operating environment. Therefore, the proposed routing protocol not only improves the life cycle of the sub-network, but also increases the amount of data received by the sink node, which would provide abundant data and information support for the stable operation of the railway monitoring system.

**Figure 11:**
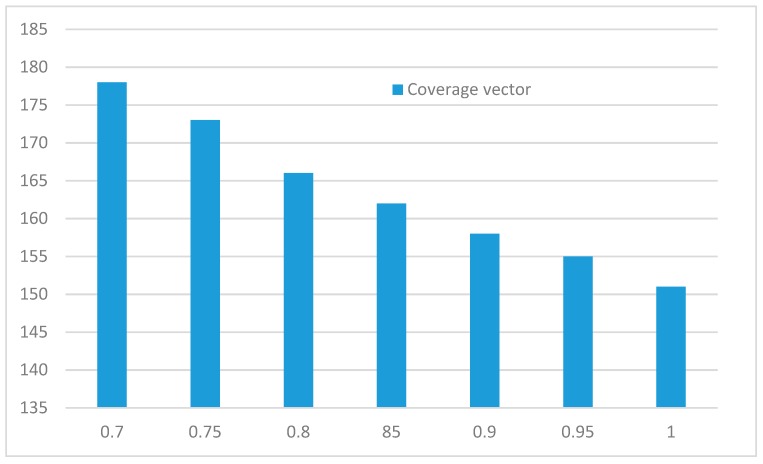
Relationship of coverage vector and network lifetime.

For partial coverage model, a total of 200 sensor nodes were randomly distributed in the monitoring area, the initial energy of wireless sensor node was 100, the number of target was 40. The initial value of coverage vector θ was 0.7, vector step was 0.05, the final value was 1. The simulation result is shown in Figure 11.

As shown in Figure 11, the lifetime of the network decreases linearly with the increase of partial coverage vector in fixed step. It shows that the coverage vector is inversely proportional to the network lifetime. In the process of node $s_{k}$ monitoring target $r_{k}$, the increase of fixed step of coverage vector is equivalent to the linear increase of coverage time, so the coverage time of node *s* increases, it leads to the increase of energy consumption, so the lifetime of network decreases linearly.

## 4. Discussion and Conclusions

The special wireless sensor network for railway status monitoring is dedicated to real-time acquisition of service status information of railway infrastructure and its operating environment, and it can rapidly transmit monitoring data to remote data centers for the diagnosis and prediction of the service status of the system. However, due to the limited energy storage of each node in the special wireless sensor network, improving the energy efficiency of the nodes in the network and prolonging the lifetime of the network are the focus of the research.

Many researchers begin with the design and optimization of routing protocols in wireless sensor networks for status monitoring [25,26,27]. In this paper, we designed a double-layer communication network based on wireless sensor network for intelligent railway systems firstly. In the sub-network, routing protocol optimization strategy are adopted to optimize the allocation of energy resources, so as to improve the efficiency of energy resources utilization of the system and maximize the lifecycle of special wireless sensor networks.

Compared with other existing methods, we proposed cluster head selection and initialization method based on improved K-medoids algorithm, which greatly improves the speed and effect of cluster optimization. At the same time, the proposed clustering optimization model is dynamic, it can make the sub-network maintain the optimal energy consumption for monitoring data transmission, so as to ensure that each cluster head node always works in the optimal energy consumption state.

Although we had done some related work in energy optimization of railway wireless monitoring system, there are also limitations. First, the research in this paper is based on local information acquisition. However, railway systems are complex and huge systems. Single local information is far from enough for evaluating the overall performance of the system and predicting the overall security situation of the system. Second, the results of optimization in this paper are mainly validated based on laboratory data and simulation data. In order to meet the needs of field application, the next step is to collect actual monitoring data. Third, multi-objective optimization model is established to solve the optimization of energy resources utilization in this paper. In the future we will make full use of the historical data of railway systems and synthesize the machine learning method to solve the optimization problem for satisfying the real-time demand. 

## Figures and Tables

**Table 1:** Simulation parameters of routing protocol based on clustering optimization.

Parameter	Description	Value
$E_{el}$	Circuit energy consumption parameter	45
$\rho_{f}$	Free space energy consumption parameters ($d^{2}$)	8
$\rho_{m}$	Multipath fading energy consumption parameter ($d^{4}$)	0.0012
$E_{i}$	Initial energy	0.02
*T*	Number of sensor nodes	200
*l*	Packet size	100
$k_{opt}$	Number of clusters	8
*m*	Population size	100
$p_{m}$	Mutation rate	0.015
$p_{c}$	Crossover rate	0.8
*t*	Maximum number of iterations	400
α	Position adjustment factor of sink node	0.5
